# Physical Plasma-Treated Skin Cancer Cells Amplify Tumor Cytotoxicity of Human Natural Killer (NK) Cells

**DOI:** 10.3390/cancers12123575

**Published:** 2020-11-30

**Authors:** Ramona Clemen, Pepijn Heirman, Abraham Lin, Annemie Bogaerts, Sander Bekeschus

**Affiliations:** 1 ZIK plasmatis, Leibniz Institute for Plasma Science and Technology (INP), Felix-Hausdorff-Str. 2, 17489 Greifswald, Germany; ramona.clemen@inp-greifswald.de (R.C.); pepijn.Heirman@student.uantwerpen.be (P.H.); 2 Research group PLASMANT, Department of Chemistry, University of Antwerp, Universiteitsplein 1, 2610 Antwerpen-Wilrijk, Belgium; abraham.lin@uantwerpen.be (A.L.); annemie.bogaerts@uantwerpen.be (A.B.); 3 Center for Oncological Research (CORE)–Integrated Personalized & Precision Oncology Network (IPPON), University of Antwerp, Universiteitsplein 1, 2610 Antwerpen-Wilrijk, Belgium

**Keywords:** kINPen, NK-cells, plasma medicine, reactive oxygen species, ROS

## Abstract

**Simple Summary:**

Natural killer (NK)-cells are known to have antitumor potential. Cold physical plasma generates ROS exogenously to be utilized as a novel anticancer agent, especially in skin cancer. However, it is unknown whether plasma-treated skin cancer cells promote or inhibit NK-cell-mediated toxicity. To this end, we analyzed NK-cell-activating receptors on plasma-treated skin cancer cells and demonstrated an enhanced NK-cell activity augmenting tumor cell death upon plasma treatment.

**Abstract:**

Skin cancers have the highest prevalence of all human cancers, with the most lethal forms being squamous cell carcinoma and malignant melanoma. Besides the conventional local treatment approaches like surgery and radiotherapy, cold physical plasmas are emerging anticancer tools. Plasma technology is used as a therapeutic agent by generating reactive oxygen species (ROS). Evidence shows that inflammation and adaptive immunity are involved in cancer-reducing effects of plasma treatment, but the role of innate immune cells is still unclear. Natural killer (NK)-cells interact with target cells via activating and inhibiting surface receptors and kill in case of dominating activating signals. In this study, we investigated the effect of cold physical plasma (kINPen) on two skin cancer cell lines (A375 and A431), with non-malignant HaCaT keratinocytes as control, and identified a plasma treatment time-dependent toxicity that was more pronounced in the cancer cells. Plasma treatment also modulated the expression of activating and inhibiting receptors more profoundly in skin cancer cells compared to HaCaT cells, leading to significantly higher NK-cell killing rates in the tumor cells. Together with increased pro-inflammatory mediators such as IL-6 and IL-8, we conclude that plasma treatment spurs stress responses in skin cancer cells, eventually augmenting NK-cell activity.

## 1. Introduction

The immune system protects the body from pathogens and the damage they inflict. It is classically divided into innate immunity and adaptive immunity. The local inflammatory reaction of myeloid cells carries out an early, innate immune response. They secret cytokines and chemokines to recruit immune cells such as natural killer cells (NK-cells) circulating in the blood. Evidence has shown that reactive oxygen species (ROS) are involved in inflammation, immune cell activation, and the modulation of the tissue microenvironment [1]. Similar findings were made in the tumor microenvironment [2], making ROS a putative target and treatment strategy in oncology [3].

Several technologies exploit the generation of ROS as anticancer agents, such as photodynamic therapy [4]. Another potent ROS-generating technology emerging as an anticancer tool is cold physical plasma [5]. This partially ionized gas is operated at body temperature and therefore does not inflict thermal damage within the target tissue. Several plasma technologies are accredited as medical products in Europe [6]. One type of plasma source is the atmospheric pressure plasma jet. Here, the working principle includes using a noble gas ionized within a high-frequency electrode shielded by a dielectric barrier [7]. The highly energetic noble gas molecules are subsequently expelled to the ambient air, reacting with oxygen and nitrogen to form reactive oxygen and nitrogen species, respectively [8]. With hundreds of chemical reactions taking place in the millisecond range, the redox chemistry in those plasma systems is highly complex and still subject to investigation [9,10]. Physical plasma treatment was shown to promote cell death in several tumor cell types, including skin cancer [11,12,13,14]. Additionally, we have previously shown an increased surface expression or release of calreticulin, ATP, and heat-shock proteins in plasma-treated tumor cells [15,16,17]. These molecules are important for triggering the activation of innate immune cells and the immunogenic cancer cell death (ICD) [18]. Subsequently, this can lead to enhanced immuno-protection upon plasma treatment in vivo [19,20,21]. However, the consequences of plasma-induced cancer cell death perceived by other types of innate immune cells, such as NK-cells, is unexplored.

NK-cells are lymphocytes and part of the innate immune system [22]. They can recognize virus-infected cells, tumor cells, and immunoglobulin-labeled cells [23] that are subsequently either ignored or lysed [24,25]. Unlike T-cells, which have specific receptors to interact with peptide-antigens presented by major histocompatibility complexes (MHC), NK-cells express germline-encoded receptors. Those receptors recognize evolutionary-conserved target structures expressed on the surface of infected, stressed, and malignantly transformed cells. To circumvent T-cell mediated tumor cell removal, cancer cells can downregulate MHC class I (“missing self”). This, however, leads to the activation of NK-cells since regular MHC-I expression inhibits NK-cell activity. Another hallmark of malignant transformation is the expression of stress-induced ligands (“stress-induced self”) on the cell surface, which also activates NK-cells by binding to receptors on the NK-cell surface [22,24]. These include NKG2D that binds to stress-induced ligands of the MHC class I chain-related proteins (MIC A and MIC B; MIC A,B). Inhibiting NK-cell receptors recognize different types of MHC class I, i.e., various kinds of the killer immunoglobulin-like receptor (KIR) and the heterodimer of CD94 (NKG2A) [26,27]. Finally, the immune checkpoint receptor PD-1 is also expressed by NK-cells, resulting in a robust inhibitory signal upon binding to its ligand CD274 (PD-L1), frequently overexpressed on cancer cells [28]. Thus, NK-cell activation is triggered upon tipping the balance of inhibitory to activating signals on the target cell surface. Once NK-cells are activated, they secrete cytokines and release cytotoxic granule enzymes to initiate tumor cell killing [29].

In this study, we investigated the cytotoxic effects of cold physical plasma on two human skin cancer cell lines that were subsequently co-cultured with human peripheral blood-derived NK-cells. Using HaCaT keratinocytes as a non-malignant control cell line, we identified plasma treatment to potentiate NK-cell-mediated inactivation of tumor cells and to spur the release of inflammatory mediators.

## 2. Results

### 2.1. Plasma Treatment Inactivated Skin Cancer Cells and Modulated NK-Cell Ligand-Receptor Expression

**Figure 1:**
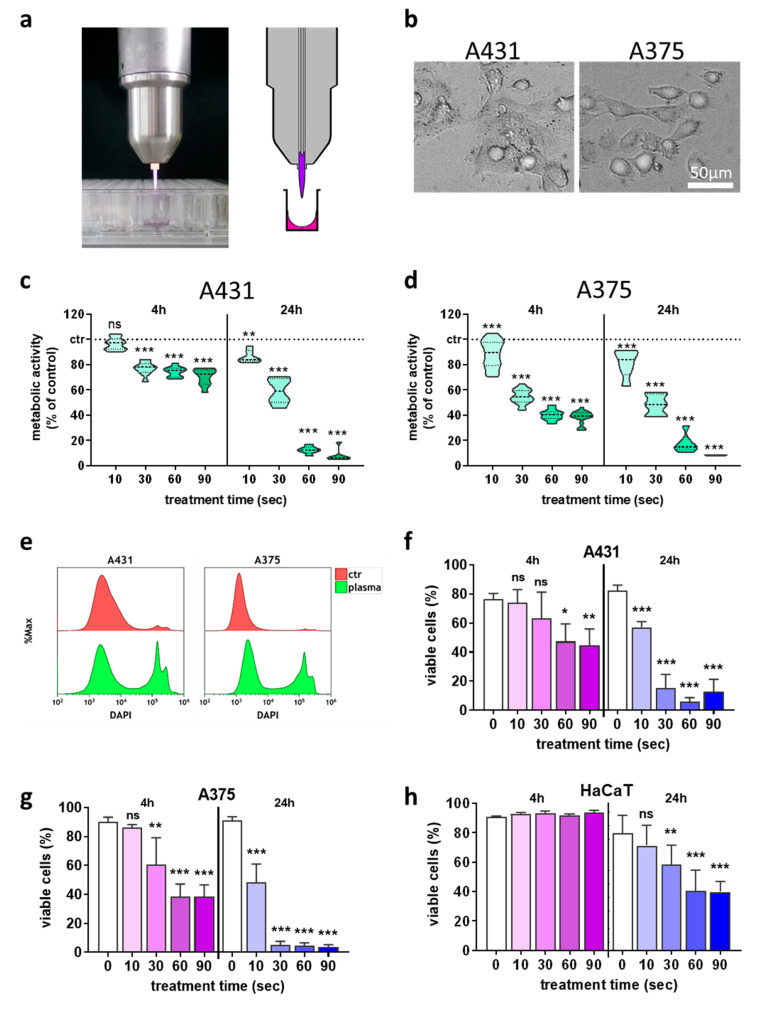
Plasma treatment inactivated skin cancer cells in a treatment time-dependent fashion. (**a**) atmospheric pressure argon plasma jet kINPen; (**b**) representative brightfield image of the two cancer cell types used in this study; (**c**,**d**) normalized metabolic activity 4 h and 24 h after plasma treatment in A431 (**c**) and A375 (**d**) cells; (**e**) representative overlay flow cytometry histograms of the terminal cell death dye DAPI in cells with and without plasma treatment at 24 h; (**f**–**h**) quantification of viable A431 (**f**), A375 (**g**), and HaCaT (**h**) cells using flow cytometry. Data are the mean of three independent experiments. Statistical analysis was performed using one-way ANOVA (* = *p* < 0.01, ** = *p* < 0.01, *** = *p* < 0.001). ns = not significant, ctr = control.

We aimed at investigating the consequences of plasma-inactivated tumor cells on NK-cell activity. To this end, the effect of plasma treatment (Figure 1a) on the skin cancer cells (Figure 1b) alone was investigated first to identify suitable dose regimens using the kINPen plasma jet. Plasma treatment decreased the metabolic activity of the skin cancer cell lines A431 (Figure 1c) and A375 (Figure 1d) in a treatment time-dependent manner. To analyze the percentage of dead cells, flow cytometry was used (Figure 1e), which confirmed the cytotoxic effects in A431 (Figure 1f) and A375 (Figure 1g) cells 24 h after plasma treatment. Importantly, plasma-induced cytotoxicity in non-malignant HaCaT cells (Figure 1h) was much less pronounced when compared to results observed in the skin cancer cells (Figure 1f,g).

**Figure 2:**
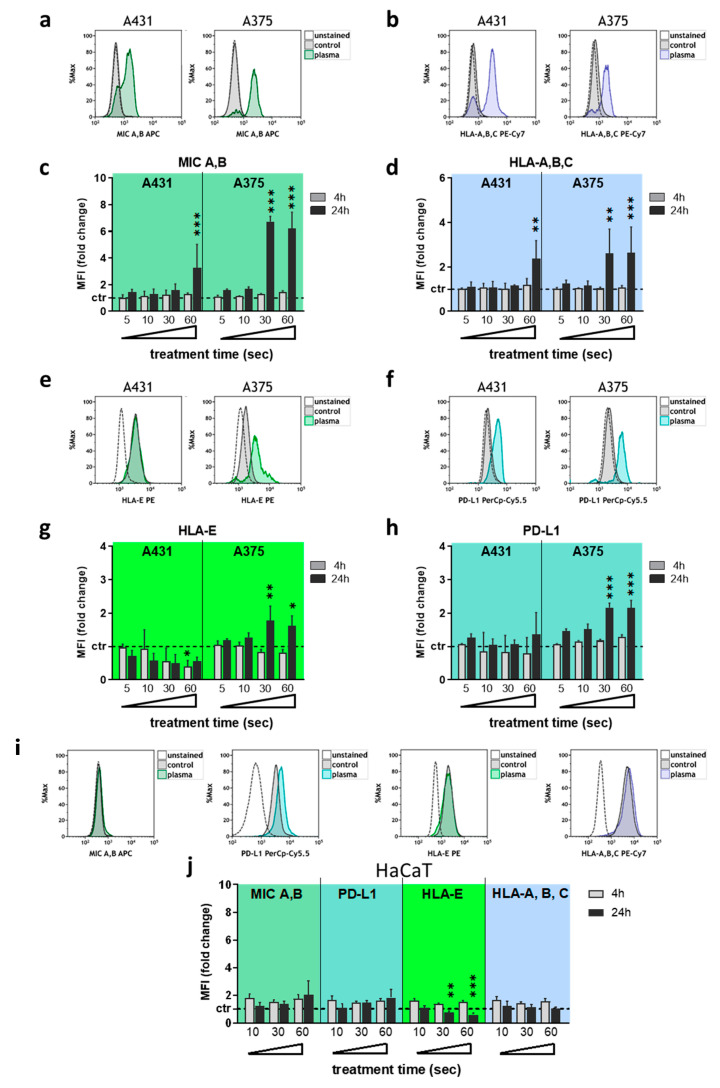
Plasma treatment modulated NK-cell ligand-receptor expression predominantly in malignant cells**.** (**a**–**d**) representative overlay flow cytometry histograms of MIC A,B (**a**) and HLA-A,B,C (**b**), and quantification and normalization of the MFI of MIC A,B (**c**) and HLA-A,B,C (**d**) in viable A431 and A375 cells at 4 h and 24 h after plasma treatment; (**e**–**h**) representative overlay flow cytometry histograms of HLA-E (**e**) and PD-L1 (**f**), and quantification and normalization of the MFI of HLA-E (**g**) and PD-L1 (**h**) in viable A431 and A375 cells at 4 h and 24 h after plasma treatment; (**i,j**) representative overlay flow cytometry histograms of MIC A,B, PD-L1, HLA-E, and HLA-A,B,C (**i**) and quantification and normalization of their corresponding MFI (**j**) in viable HaCaT cells at 4 h and 24 h after plasma treatment. Data are the mean of three independent experiments. Statistical analysis was performed using one-way ANOVA (* = *p* < 0.01, ** = *p* < 0.01, *** = *p* < 0.001). MFI = mean fluorescent intensity.

Next, the surface expression of several NK-cell-relevant ligands was investigated at 4 h and 24 h after plasma treatment using multi-color flow cytometry. The analysis of MIC A,B (Figure 2a) and HLA-A,B,C (Figure 2b) revealed a significant increase of the former (Figure 2c) and the latter (Figure 2d) in both A431 and A375 cells at 24 h post plasma exposure for extended treatment times. For HLA-E (Figure 2e) and PD-L1 (Figure 2f), a significant decrease in A431 and increase in A375 was found for HLA-E expression (Figure 2g), while no change was found in A431 for PD-L1 (Figure 2h). In A375, PD-L1 was upregulated when exposed to extended plasma treatment times. Only viable (DAPI^-^) cells were used for data analysis, and no relevant changes were found in either of the cell lines at 4 h after plasma treatment. To compare these results against a non-malignant cell line, HaCaT keratinocytes were investigated for their expression of the same molecules following plasma exposure (Figure 2i). Besides a decrease in HLA-E at 24 h, no significant changes were observed for any of the remaining targets investigated (Figure 2j).

Altogether, a plasma treatment time-dependent cytotoxicity was observed in the skin cancer cell lines A375 and A431, while non-malignant HaCaT keratinocytes were less affected. At longer plasma treatment times, a significant modulation of NK-cell-relevant ligands was observed on the tumor cells’ surface. As we aimed at investigating the crosstalk of viable tumor cells with human NK-cells to allow investigating additive toxicity, a moderate plasma treatment time (10 s) was used for subsequent co-culture experiments. In plasma-killed tumor cells, increased MIC A,B expression associated with stress responses was also found for plasma treatment times shorter than 60 s in A431 (Figure S1a) and A375 (Figure S1b) cells at 24 h.

### 2.2. Plasma-Treated Tumor Cells Augmented NK-Cell-Mediated Toxicity

**Figure 3:**
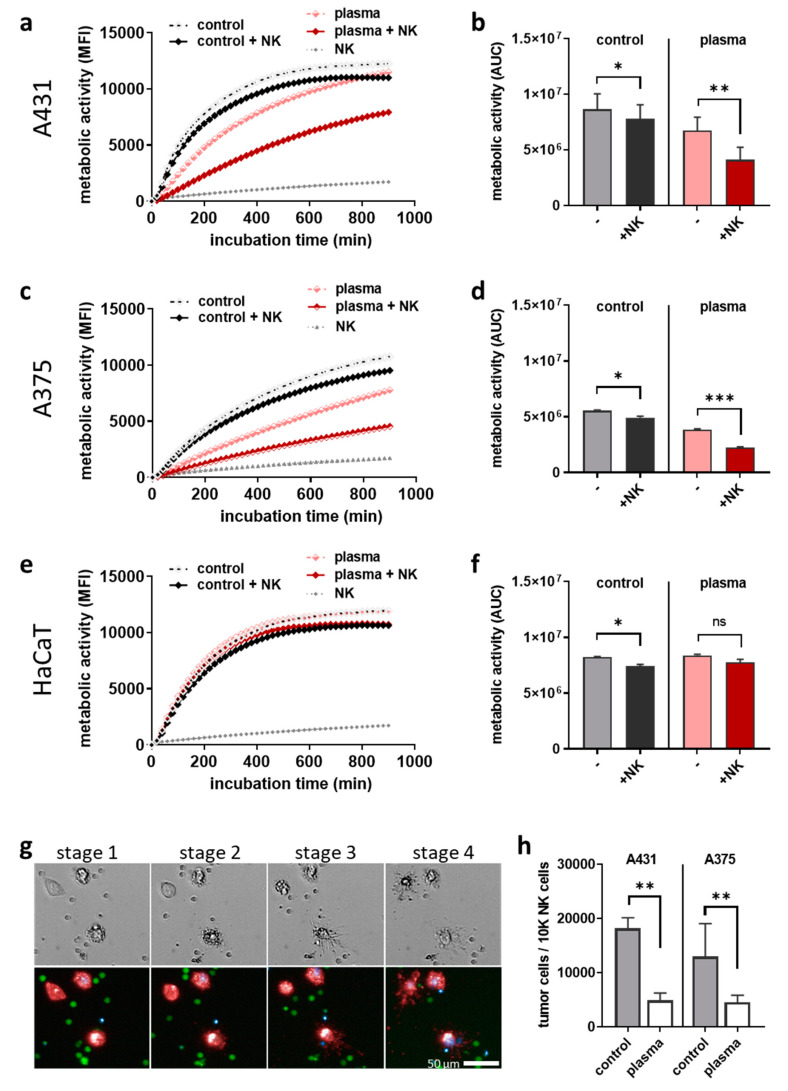
Plasma-treated tumor cells augmented NK-cell-mediated toxicity. (**a**–**f**) kinetic metabolic activity and the corresponding AUC in untreated and plasma-treated A431 (**a**,**b**), A375 (**c**,**d**), and HaCaT (**e**,**f**) cells in the absence or presence of NK-cells (effector-target-ratio 1:1); (**g**) representative brightfield (upper row) and fluorescence (lower row) images of co-culture with red-labeled tumor cells, green-labeled NK-cells, and blue-labeled dead cells; (**h**) tumor cell count normalized to NK-cell count at 24 h after initiation of co-cultures using flow cytometry. Data are the mean of three independent experiments. Statistical analysis was performed using one-way ANOVA (* = *p* < 0.01, ** = *p* < 0.01, *** = *p* < 0.001). ns = not significant, MFI = mean fluorescent intensity, AUC = area under the curve.

The question of our study was whether plasma-treated cancer cells inhibit or augment NK-cell-mediated toxicity. To this end, plasma-treated skin cancer cells were incubated for 24 h, followed by the co-culture with human NK-cells at an effector-target-ratio of 1:1. Kinetic metabolic activity assays served to investigate the cytotoxic responses. In A431 cells (Figure 3a), the addition of NK-cells caused a greater decline of A431 metabolic activity in plasma-treated cells compared to untreated tumor cells (Figure 3b). In A375 cells (Figure 3c), a similar effect was observed (Figure 3d), suggesting an enhanced NK-cell activity against the plasma-treated cancer cells. In co-cultures of NK-cells with HaCaT keratinocytes (Figure 3e), such an augmented activity was not observed upon plasma treatment (Figure 3f). Using multiparametric fluorescence microscopy, a qualitative analysis of co-cultures confirmed that the decline of metabolic activity was related to cell death, as tumor cells (stage 1, t = 5 h after addition of NK-cells) upon contact with NK-cells (stage 2, t = 6; stage 3, t = 7 h) led to rapid terminal cell death (stage 4, t = 11 h) (Figure 3g). This notion of NK-cell-enhanced cytotoxicity in plasma-treated tumor cells was supported quantitatively by calculating the tumor cell to NK-cell ratios using absolute cell counting by flow cytometry 24 h after co-culture initiation (Figure 3h). These findings collectively suggest that plasma treatment inflicted stress in A375 and A431 skin cancer cells but not non-malignant HaCaT keratinocytes, leading to the enhanced recognition and killing of the tumor cells by NK-cells.

### 2.3. Plasma-Treated Tumor Cells Stimulated the Secretion of Inflammatory Mediators upon Co-Culture with NK-Cells

**Figure 4:**
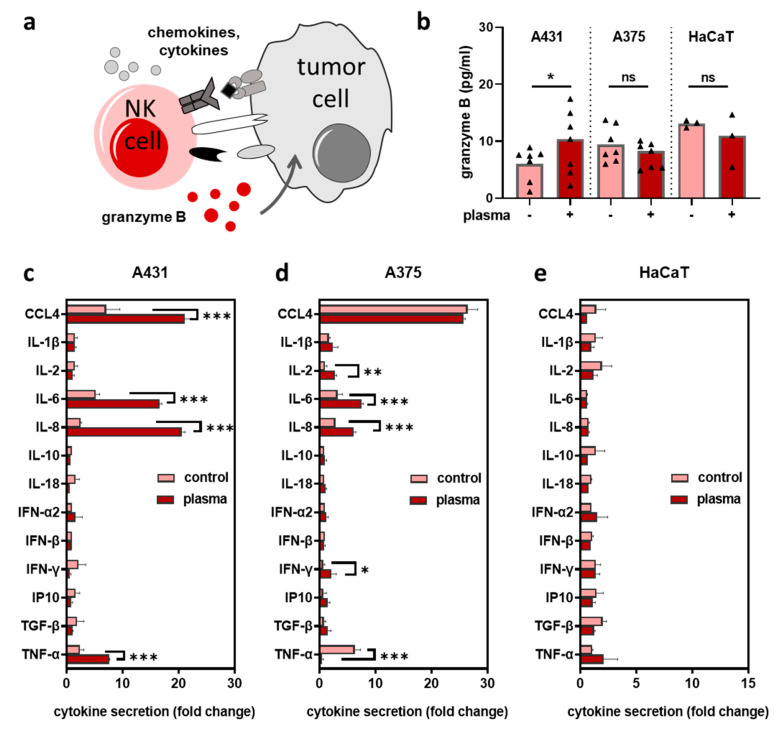
Plasma-treated skin tumor cells stimulated the secretion of inflammatory mediators upon co-culture with NK-cells. (**a**) simplified scheme and consequences of NK-cell-tumor cell interaction; (**b**) granzyme B quantification in supernatants of co-cultures (±plasma treatment) at 4 h, 6 h, and 24 h (A431 and A375) and 24 h (HaCaT); (**c**–**e**) quantification of cytokines in co-cultures in supernatants of untreated (vehicle) and plasma-treated A431 **(c**), A375 (**d**), and HaCaT (**e**) cells at 24 h as a ratio to the respective single tumor cell or HaCaT keratinocyte culture. Data are the mean of three independent experiments. Statistical analysis was performed using t-test or two-way ANOVA (* = *p* < 0.01, ** = *p* < 0.01, *** = *p* < 0.001, ns = not significant).

Activated NK-cells release cytotoxic granule enzymes to initiate killing and secret stimulatory cytokines to recruit immune cells (Figure 4a). To investigate NK-cell activation in co-cultures upon plasma treatment, we measured the concentration of granzyme B in supernatants at 4 h, 6 h, and 24 h (except for HaCaT only at 24 h). Granzyme B levels were increased in plasma-treated vs. untreated A431 cells, but not in A375 or HaCaT cells co-cultured with NK-cells (Figure 4b). Subsequently, we measured the secretion of several pro and anti-inflammatory cytokines 24 h after co-culture and shown as ratio of the co-culture concentration to the respective mono-culture concentration. A significant increase of interleukin (IL)-6 and IL-8 was observed for plasma treatment in A431 (Figure 4c) and A375 (Figure 4d) but not HaCaT (Figure 4e) cells. In general, plasma-treated HaCaT cells in co-culture with NK-cells did not show significant changes in the analytes’ levels compared to co-cultures with untreated HaCaT cells. By contrast, significant elevation of Chemokine (C-C motif) ligands (CCL) 4 and tumor necrosis factor (TNF)-α was found in plasma conditions for A431, while in A375 co-culture supernatants, plasma treatment led to a significant increase in interferon (IFN)-γ and IL-2, whereas TNF-α levels declined. These findings suggested that plasma-treated skin cancer cells provoked a pro-inflammatory milieu when encountered by NK-cells.

### 2.4. H_2_O_2_ Treatment Did Not Replicate Results Observed with Plasma Treatment

**Figure 5:**
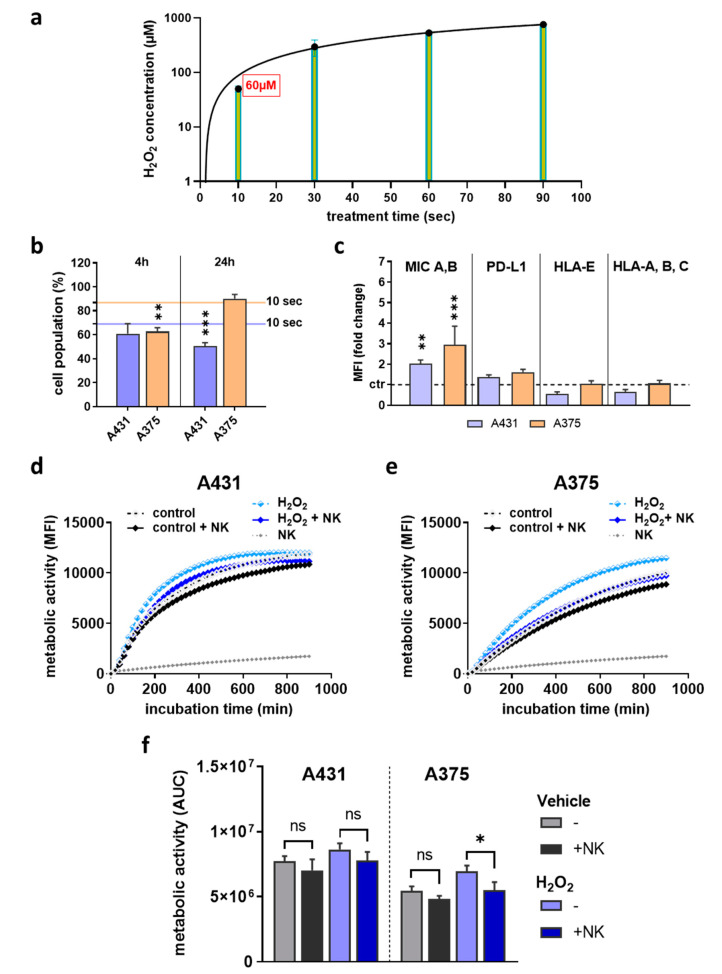
H_2_O_2_ treatment did not replicate the results observed with plasma treatment. (**a**) 10 s of plasma treatment yielded approximately 60 µM of H_2_O_2_ in solution, which was subsequently used as control treatment; (**b**) cell viability of A431 and A375 cells at 4 h and 24 h after H_2_O_2_ treatment as determined using flow cytometry; (**c**) quantification of the expression of surface markers MIC A,B, PD-L1, HLA-E, and HLA-A,B,C in viable cells 24 h after H_2_O_2_ treatment using flow cytometry; (**d**–**f**) H_2_O_2_-treated or untreated A431 (**d**) and A375 (**e**) cells in presence or absence of NK-cells, and kinetic assessment of metabolic activity as well as AUC (**f**) of the data over 15 h. Data are mean of three independent experiments. Statistical analysis was performed using one-way ANOVA or t-test (* = *p* < 0.01, ** = *p* < 0.01, *** = *p* < 0.001). ns = not significant, AUC = area under the curve, MFI = mean fluorescent intensity.

Hydrogen peroxide (H_2_O_2_) is one of the most abundant ROS produced in plasma-treated liquids [30], but it is not always clear to which extent the findings with plasma treatment depend on the generation of this agent. Accordingly, the plasma generated H_2_O_2_ levels were quantified, and the concentration of 60 µM was equivalent to the 10 s of plasma treatment time used in the previous experiments (Figure 5a). The treatment of skin cancer cells with this concentration generated somewhat more cytotoxic responses in A431 after 24 h and A375 after 4 h (Figure 5b) compared to the corresponding plasma treatment time (solid lines). When investigating the ligand expression (Figure 5c), only MIC A,B differed significantly after H_2_O_2_ treatment. Kinetic metabolic activity assessment of NK-cells co-cultured with H_2_O_2_-treated A431 (Figure 5d) or A375 (Figure 5e) cells suggested H_2_O_2_ treatment to spur metabolic activity in skin cancer cells (Figure 5f), which is in stark contrast to findings with low-dose plasma treatment. The addition of NK-cells caused a reduced metabolic activity, suggesting some degree of cytotoxicity against the tumor cells. However, the amplitude of differences between samples with or without NK-cells and between untreated and H_2_O_2_-treated cells was smaller than in the plasma treatment regimens. These results suggested that H_2_O_2_ treatment yielded partially similar but not identical results in terms of cytotoxicity, surface marker expression, and consequences of co-culture in vitro with human NK-cells.

## 3. Discussion

In plasma medicine, oncology is a promising research field, and successful tumor reduction has already been observed in animal models and human patients suffering from endstage head and neck cancer [31,32]. Moreover, the exposure of cancer tissue to cold physical plasma was recently outlined to have an immunological dimension [33], but NK-cells have not been studied in the field of plasma medicine so far. To this end, we here investigated three human cell lines (two of malignant origin) and human peripheral blood-derived NK-cells to understand the immunomodulating consequences of plasma-treated skin cancer cells in co-culture with NK-cells

Plasma-treated skin cancer cells augmented NK-cells-mediated tumor cell inactivation. It is known that plasma jets, such as the kINPen, expel a plethora of ROS simultaneously [34] that, in an exogenous manner, subsequently target cells. Interestingly, the increase of surface marker expression, as, e.g., MIC A,B in response to extracellular ROS, has been described previously [35,36]. Investigating intracellular ROS generation using pharmacological (sulforaphane) or physical (ionizing radiation) agents was previously found to enhance NK-cell-mediated tumor cell lysis using several cancer cell lines [37]. Similar to our findings, the authors found an increase of the NKG2D ligand MIC A,B in tumor cells upon intracellular ROS increase. With plasma treatment, it is also established that extracellular ROS generation subsequently raises intracellular ROS levels [38]. Another study using sublethal doses of hematoporphyrin-based photodynamic therapy reached a similar conclusion based on elevated MIC A,B tumor cell expression in the context of ROS generation, and subsequently enhanced NK-cell-mediated killing [39]. However, it needs to be stressed that ROS severely impair NK-cell activity and survival, as IL-15-primed NK-cells upregulate thioredoxin activity to protect themselves from cytotoxic ROS in the tumor microenvironment (TME) [40]. This is in line with the notion that NK-cells are sensitive against ROS like hydrogen peroxide [41]. These aspects were incorporated in our study design by adding NK-cells to the plasma-treated tumor cells only at 24 h post-exposure, allowing the plasma-introduced ROS to deteriorate before NK-cell addition.

Besides MIC A,B, we have also investigated other effectors with a putative contribution to our findings. This includes HLA-A,B,C that can trigger NK-cell-activation when absent via KIR receptor ligation [42], but HLA-A,B,C expression was not substantially altered for the moderate plasma treatment time (10 s) investigated. HLA-E, an inhibitory ligand signaling via CD94/NKG2A, was found to be decreased upon selenite-induced oxidative stress and facilitated the enhanced NK-cell-mediated killing of tumor cells [43]. Moderate plasma treatment downregulated HLA-E in A431 but not A375 cells, which might be linked to the increased levels of granzyme B in supernatants of the co-culture with the former over the latter. For PD-L1, a potent NK-cell inhibitor upon ligation of PD-1 [44], plasma treatment showed no substantial increase at shorter treatment times, making its contribution to a more-than-average inhibition of NK-cells unlikely. In supernatants of co-cultures of NK-cells with either of the skin cancer cell lines but not HaCaT keratinocytes, we identified elevated IL-6 and IL-8 levels in plasma conditions. IL-8 was previously shown to be critical for NK-cell chemotaxis [45] and might have contributed to guide NK-cells to the tumor cells on a microscale in the co-culture conditions. Nevertheless, at least in colorectal cancer, the positive protective value of IL-8 and granzyme B levels in the TME is related to T-cells rather than NK-cells [46]. Due to multiple cell types producing IL-6 and its pleiotropic effects [47], an unambiguous role of the increased levels of IL-6 in the plasma conditions cannot be identified.

Studies using the kINPen so far have concluded that non-malignant tissue is affected only to a minor extent [48], with no adverse long-term side effects and including increased metastasis [49,50,51], and a lack of genotoxicity of the plasma treatment procedure [52,53]. The present study demonstrated that prolonged exposure to plasma drastically decreases viability and metabolic activity in two skin cancer cell lines. In contrast, the viability of non-malignant skin cells treated under the same conditions was far less affected. Changes in chemokine and cytokine release were observed for NK-cell co-cultures with skin cancer cells. Conversely, plasma treated non-malignant HaCaT cells showed neither an altered marker expression after plasma treatment nor an altered metabolism or cytokine profile in co-culture with NK-cells. Both effects confirmed our hypothesis and indicated an immune-stimulating and tumor-specific impact of the plasma treatment as previously suggested and independent of NK-cells [54,55].

While we could not identify changes in one surface molecule that facilitated improved tumor surveillance, our data suggest several effectors at play. The NK-cell activation after target-cell engagement binding is determined by a balance between both activating and inhibiting signals. Our data suggest MIC A,B to be of paramount importance, as its role in NK-cell activation is well described, and its upregulation was found in our study. However, it cannot be solely responsible for the effects observed as it was also increased in plasma-treated HaCaT cells, which did not promote NK-cell activation. Besides, the fact that H_2_O_2_ treatment did not replicate the plasma effects supports the conclusion that other types of plasma-derived ROS or RNS [56] might be involved in explaining our findings. A likely possibility is that the plasma treatment modulated the expression of ligands other than the ones investigated in this study. A wide range of ligands and receptors contribute to the interaction between NK-cells and tumor cells [28]. This, together with exploring the role of NK-cells in plasma treatment in experimental tumor models in vivo, should be deciphered in future studies in more detail.

In our study, the epidermoid carcinoma A431 and the human melanoma cell line A375 were used, each with distinct activation pathways. A431 express abnormally high levels of the epidermal growth factor (EGF) receptor but are void of the tumor suppressor p53 [57]. The cells can differentiate through the JNK pathway [58]. As non-malignant cell line, we used HaCaT keratinocytes. The ROS treatment of those cells induced p53 and JNK phosphorylation as well as MAPK activation [59]. As for similarities, it was found that IFN-γ treatment induces MHC-I upregulation in both A431 and non-malignant keratinocytes [60]. Moreover, UVB treatment increases sestrin-1 in both HaCaT and A431 cells [61], which promotes AKT activation through a PTEN-related mechanism. The amelanotic A375 cells also harbor PTEN but differ from A431 as well as HaCaT cells in multiple ways, such as a highly aberrant expression of the putative oncogenic transcription factor NFAT [62] and the mutation profile (Figure S1c). Nevertheless, the plasma treatment showed overall similar effects in both cell lines, suggesting that their responses to oxidative stress might be related. The triad of A431, A375, and HaCaT keratinocytes has been used in multiple studies before demonstrating similar effects in A431 and A375 [63,64,65,66]. However, a limitation of our study was the lack of non-malignant primary melanocytes as a control for melanoma. Hence, the selectivity of the plasma treatment towards A375 cells could not be determined. Notwithstanding, it needs to be mentioned that the plasma therapy is a local, topical treatment. Other local treatments, such as cryoablation, photodynamic therapy, and electrochemotherapy, also come with a certain degree of collateral damage to non-malignant cells in the TME but still have been proven to be clinical efficacious in several cancer types [67,68,69]. Regardless, previous studies using other plasma devices have provided evidence of a selective toxicity of plasma treatment in malignant melanoma cells over non-malignant melanocytes [70,71], underlining the findings for non-melanoma skin cancer and HaCaT keratinocytes in the present study.

## 4. Experimental Section

### 4.1. Cell Culture and NK-Cell Isolation

The human epidermoid carcinoma cell line A431 (ATCC CRL-1555), the malignant melanoma cell line A375 (ATCC CRL-1619), and the non-malignant HaCaT keratinocyte cell line (CVCL-0038) were cultured in Roswell Park Memorial Institute (RPMI 1640; Corning, Kaiserslautern, Germany) medium containing 10% fetal bovine serum (Sigma-Aldrich, Hamburg, Germany), 1% glutamine (Corning), and 1% penicillin/streptomycin (Corning). The cells were grown in tissue-culture treated cell culture flasks (Sarstedt, Sarstedt, Germany) at 37 °C, 95% humidity, and 5% CO_2_, and subcultured twice a week. Peripheral blood was obtained with informed consent from healthy donors as approved by the local ethics committee (approval number BB166/17). Peripheral blood mononuclear cells were isolated as described before [72] via the Ficoll-Paque density gradient centrifugation method. Erythrocytes were lysed (RBC lysis buffer; BioLegend, Amsterdam, The Netherlands), and CD56^+^ NK-cells were negatively selected via magnetic bead separation (BioLegend). The cells were washed and resuspended in fully supplemented cell culture medium.

### 4.2. Plasma Jet Treatment

Plasma treatment was performed using the atmospheric pressure plasma jet kINPen (neoplas, Greifswald, Germany) and argon (purity 99.9999%; Air Liquide, Paris, France) as carrier gas at a flow rate of two standard liters per min. The jet is extensively characterized [8], and the pin-type powered electrode was operated in a dielectric ceramic tube (inner diameter: 1.6 mm; outer diameter: 2.0 mm) with a grounded electrode at a frequency of 1 MHz. The distance from the exit to the nozzle tip is 3.5 mm. It has a dissipated power of 3.5 W. For the plasma treatment, 1 × 10^4^ cells in 100 µL of fully supplemented cell culture medium were seeded in 96-well plates (Eppendorf, Hamburg, Germany) and treated with plasma in a standardized manner as described before [73]. For co-culture experiments, the medium of plasma-treated tumor cells was removed at 24 h, and 100 µL suspension containing 10^4^ NK-cells was added to each well.

### 4.3. Metabolic Activity

Metabolic activity was measured in a multimode plate reader (Tecan, Männedorf, Switzerland) at λ_ex_ 535 nm and λ_em_ 590 nm, 4 h after resazurin (Alfa Aesar, Haverhill, MA, USA) was added to the cells at a final concentration of 100 µM. Resazurin was added directly after plasma treatment to determine metabolic activity at 4 h, or after 20 h to determine metabolic activity at 24 h. For co-culture experiments, resazurin was added, and kinetic measurements were performed in a multiplate plate reader heated to 37 °C and continuously flushed with 5% CO_2_. Fluorescence was measured every 20 min over 15 h. To avoid excessive evaporation during this period, the outer cavity in the 96-well plate was filled with 6 mL of deionized water.

### 4.4. Flow Cytometry

Flow cytometry experiments were performed using a CytoFLEX LX device (Beckman-Coulter, Krefeld, Germany). Cell viability was determined using *CellEvent* Caspase 3/7 green detection agent (Thermo Fisher Scientific, Bremen, Germany) and 4′,6-diamidino-2-phenylindole dihydrochloride (DAPI; BioLegend). Surface marker expression was investigated by incubating the cells with fluorochrome-conjugated antibodies (Table 1). Data analysis was performed using Kaluza 2.1 (Beckman-Coulter).

### 4.5. High Content Imaging

The imaging of co-cultures was performed using a high content imaging system (Operetta CLS; PerkinElmer, Hamburg, Germany) equipped with a 16-bit 4.7MP sCMOS camera and a 785 nm laser autofocus. After plasma treatment, skin cancer cells were stained with the red cell labeling dye Vybrant DiD (Invitrogen; Carlsbad, CA, USA) for 90 min at 37 °C and washed with PBS. The NK-cells were stained green by incubation in RPMI containing 100 nM Calcein AM (Invitrogen) for 30 min. A washing step was done before adding the NK-cells to the cancer cells with an effector-target-ratio of 1:1. Sytox blue dead cell stain (final concentration 0.5 µM; Invitrogen) was added to each well. 96-well plates (Eppendorf) were used to facilitate imaging via a 20× water immersion objective (NA 1.0; Zeiss, Jena, Germany). Excitation and emission settings were λ_ex_ 475 nm and λ_em_ 548 ± 32 for Calcein AM, λ_ex_ 550 nm and λ_em_ 610 ± 40 for DiD red, and λ_ex_ 405 nm and λ_em_ 493 ± 23 for Sytox blue, respectively.

### 4.6. H_2_O_2_ Measurements

The concentration of H_2_O_2_ in plasma-treated medium without cells was measured using the Amplex Ultra Red reagent kit (Thermo Fisher Scientific) as described before [74].

### 4.7. Cytokine Measurement

Supernatants of single and co-cultured cells were collected after 24 h. Granzyme B secretion was measured using ELISA according to the manufacturer’s instructions (BioLegend). Parallel quantification of a set of cytokines and chemokines was done using flow cytometry (CytoFLEX S; Beckman-Coulter) and LegendPlex technology (BioLegend) as described before [75].

### 4.8. Statistical Analysis

Graphing and statistical analysis were performed using *Prism* 9.0 (GraphPad Software, San Diego, CA, USA). Comparison of two groups was made using Student’s t-test. The comparison of more than two groups was made using one-way analysis of variances (ANOVA). The comparison of more than two groups across different data sets was made using two-way ANOVA. Levels of significance were indicated as follows: α = 0.05 (*), α = 0.01 (**), α = 0.001 (***).

## 5. Conclusions

Plasma-treated tumor cells augment NK-cell activity through the modulated expression of activating and inhibiting receptors. In comparison, plasma-treated HaCaT keratinocytes also showed altered expression but did not increase NK-cell activity.

## Figures and Tables

**Table 1:** Antibodies used in this study.

Ligand	Fluorochrome	Clone	Supplier
MIC A,B	APC	6D4	BioLegend
HLA-A,B,C	PE-Cy7	G46-2.6	BD Biosciences
HLA-E	PE	3D12	BioLegend
PD-L1	PerCP/Cyanine5.5	29E2A3	BioLegend

## Supplementary Materials

The following are available online at https://www.mdpi.com/2072-6694/12/12/3575/s1, Figure S1: (a,b) surface marker expression on dead A431 (a) and A375 (b) cells 4 h and 24 h after exposure to plasma as determined using flow cytometry; (c) VENN diagram of mutations in A431 cells as compared to A375 cells as retrieved from https://portals.broadinstitute.org/ccle. Data are mean of three independent experiments. Statistical analysis was performed using one-way ANOVA (* = *p* < 0.01, ** = *p* < 0.01, *** = *p* < 0.001). MFI = mean fluorescent intensity.
