# Supplementary material for: Physical Plasma-Treated Skin Cancer Cells Amplify Tumor Cytotoxicity of Human Natural Killer (NK) Cells
Source: Cancers (Basel). 2020 Nov 30;12(12):3575. doi: 10.3390/cancers12123575 (PMC7761052; doi:10.3390/cancers12123575)
Supplement: Supplementary file 1 [file cancers-12-03575-s001.pdf]

Supplementary Materials:

Physical Plasma-Treated Skin Cancer Cells Amplify Tumor Cytotoxicity of Human Natural Killer (NK) Cells

Ramona Clemen, Pepijn Heirman, Abraham Lin, Annemie Bogaerts and Sander Bekeschus

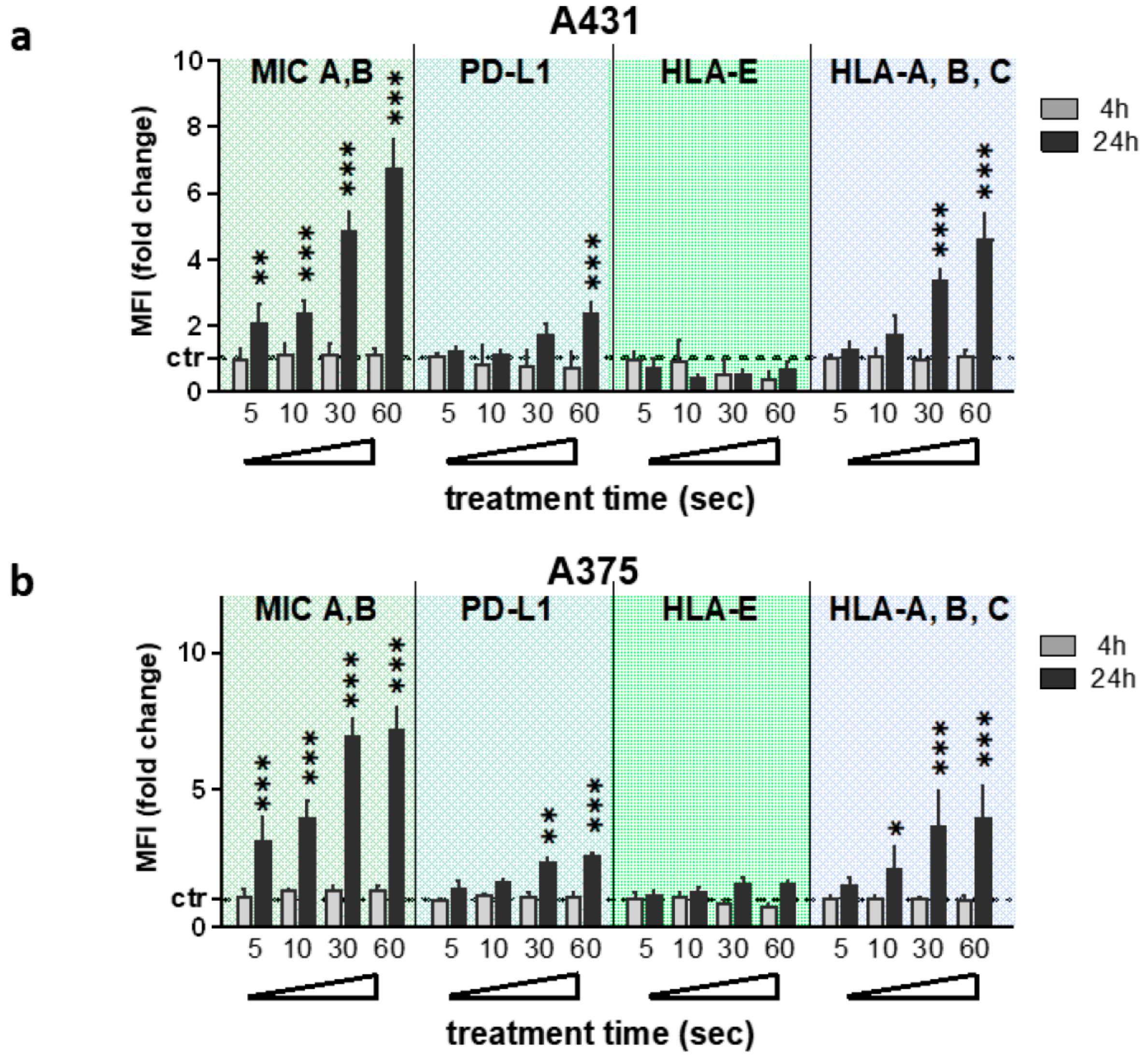

c

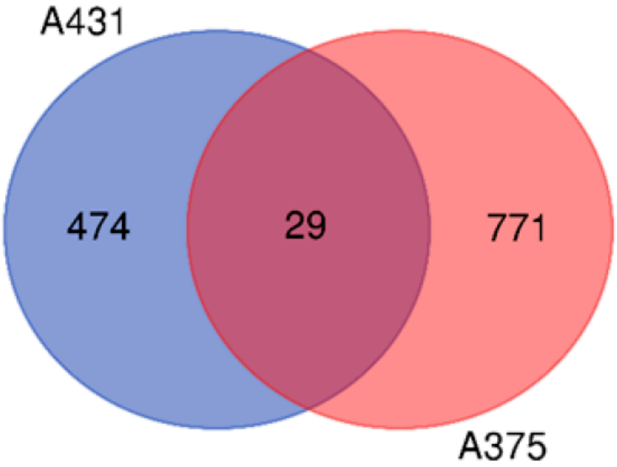

Figure S1. (**a**,**b**) surface marker expression on dead A431 (a) and A375 (b) cells 4h and 24h after exposure to plasma as determined using flow cytometry; (**c**) VENN diagram of mutations in A431 cells as compared to A375 cells as retrieved from https://portals.broadinstitute.org/ccle. Data are mean of three independent experiments. Statistical analysis was performed using one-way ANOVA (* = $p <$ 0.01, ** = $p < 0.01$, *** = $p < 0.001$). MFI = mean fluorescent intensity.
